# Discrimination, school inclusion, and quality of life in adolescence: a mediation analysis

**DOI:** 10.3389/fpsyg.2025.1722505

**Published:** 2026-01-12

**Authors:** Alba Ayuso-Lanchares, Clara González-Sangino, Jairo Rodríguez-Medina

**Affiliations:** 1 Department of Pedagogy, University of Valladolid, Valladolid, Spain; 2 Department of Psychology, University of Valladolid, Valladolid, Spain

**Keywords:** adolescents, discrimination, inclusion, quality of life, structural equation modeling

## Abstract

**Introduction:**

Discrimination is a known risk factor for poorer mental health and reduced wellbeing during adolescence. This study aims to examine whether perceived inclusion in school mediates the relationship between perceived discrimination and quality of life (QoL) among adolescents.

**Method:**

A cross-sectional study was conducted with a nationally representative sample of 839 Spanish adolescents aged 12 to 16 years. Participants completed the Everyday Discrimination Scale (EDS), the Perceptions of Inclusion Questionnaire (PIQ), and the KINDL-R QoL measure. Structural Equation Modeling (SEM) was used to analyze the direct and indirect effects of discrimination on QoL, with inclusion as a proposed mediator. Measurement invariance across gender was also assessed.

**Results:**

Perceived discrimination showed a significant negative association with QoL (*β* = −0.436, *p* < 0.001), while inclusion was positively associated with QoL (*β* = 1.575, *p* < 0.001) and negatively associated with discrimination (*β* = −0.457, *p* < 0.001). Inclusion partially mediated the impact of discrimination on QoL, with a significant indirect effect (*β* = −0.719, *p* < 0.001). Mediation models demonstrated superior fit compared to non-mediated alternatives. School inclusion mitigates the negative impact of discrimination on adolescent QoL.

**Discussion:**

Findings highlight the importance of fostering inclusive educational environments to enhance adolescent wellbeing. Future studies should examine these relationships longitudinally and across diverse cultural contexts.

## 1 Introduction

Adolescence is a stage in life development where psychosocial aspects such as the relationship with others and the need to belong are especially important (Allen et al., 2018). In this sense, the educational context is particularly relevant, as it is the place where young people spend most of their time and where many of their social interactions take place (Kiuru et al., 2020).

Inclusive settings in the educational context are key for the will-being of adolescents, as can foster a sense of belonging and acceptance, which are crucial for positive mental health development (Kyttälä et al., 2023), as well as socioemotional outcomes and academic performance (Civitillo et al., 2023). When young people feel included and supported, they are more likely to experience greater self-esteem, reduced anxiety, and a stronger sense of overall wellbeing (Boyle et al., 2023). In a recent study by Heppt et al. (2025), adolescents from ethnically minoritized groups highly stigmatized, perceived a stronger discriminatory climate and experienced classrooms as less structured and more disruptive, highlighting the mediating role of the classroom between discrimination and school adjustment. In another longitudinal study, adolescents experienced the most discrimination in the school environment, associated with decreased wellbeing and protective health behaviors, as well as increased long-term risk behaviors (Emmer et al., 2025). Inclusive education aims to promote the highest levels of presence, participation and learning for all students in the regular education system, particularly, but not only, for those in vulnerable situations (Ainscow, 2005; UNESCO, 1994; United Nations, 2006). Therefore, inclusive education means better outcomes for special needs students as well as others (de Bruin, 2019), and it is the best practice recommended by UNESCO (2005) for all students.

One of the dangers that may threaten inclusion is the presence of stigma and discrimination toward students who are considered different. Discrimination involves unequal treatment based on group or personal characteristics, indicating that others perceive you negatively and do not accept you (Uluğ and Tropp, 2021), that you are treated unfairly or that the world is unjust (Brandt and Crawford, 2020), as well as that others have control over you (Kende and McGarty, 2019). Different studies have shown the existence of discrimination during adolescence for various reasons, such as racism, gender and sexual orientation (Cave et al., 2020; Vargas et al., 2020), physical appearance or weight (Gerend et al., 2022; Sutin et al., 2020), or having a disease or a disability (Meurillon et al., 2025; Lindsay and Cao, 2025; Mulvey et al., 2025). Overall showing how adolescents who perceive greater discrimination have a lower sense of belonging to the school and community (Kilicoglu and Kilicoglu, 2024; Carnes and Disney, 2025).

In addition, it is well known that discrimination has immediate negative effects on the mental health of those who experience it (Emmer et al., 2024), as well as lasting effects on different psychosocial dimensions (Trent et al., 2019; Emmer et al., 2025). As a stressor, it can adversely affect a broad range of physical and mental health outcomes (Peterson et al., 2016; Williams et al., 2019), and coping with it can lead to an increase in risky health behaviors and a decline in the utilization of healthcare services (Williams et al., 2019). Moreover, in order to experience the negative effects of discrimination, it does not only have to be experienced, as Del Toro et al. (2024) show how in cases of witnessing ethnic-racial discrimination it is also accompanied by negative consequences, such as poor academic performance.

Given the importance of discrimination and inclusion, and their impact on the wellbeing of young people, some studies have attempted to investigate their relationships. Montoro et al. (2021), found that discrimination has a negative relationship with school belonging. Mulvey et al. (2022), found that discrimination was negatively related to inclusion and the sense of belonging, with the latter functioning as mediators between discrimination and lower engagement in the classroom. Additionally, Mulvey et al. (2021) point out the complex interaction between school and teacher factors in the face of exclusion, finding that a better school climate can promote the intention to intervene in situations of discrimination. On the other hand, they find that adolescents with previous experiences of discrimination judge it to be more acceptable (Mulvey et al., 2020) and are less likely to intervene (Mulvey et al., 2021).

Although these constructions are often discussed together in the literature, it is essential to clarify their conceptual distinctions to appropriately frame the present study. In the present study, school inclusion, sense of belonging, and wellbeing are treated as related but conceptually distinct constructs. School inclusion refers to students’ perceptions of being academically supported, socially accepted, and emotionally valued within the school environment, encompassing emotional, social, and academic dimensions (Venetz et al., 2015). Sense of belonging, by contrast, reflects the psychological experience of feeling connected, accepted, and valued as part of the school community and is widely recognized as a protective factor for adolescent development (Allen et al., 2018). Finally, wellbeing is understood as adolescents’ broader subjective and health-related quality of life, including emotional, physical, school, and social domains, as assessed through validated HRQoL instruments (Ravens-Sieberer and Bullinger, 1998; Rajmil et al., 2004). Clarifying these distinctions is essential, as previous studies have sometimes used these terms interchangeably despite their unique theoretical and functional roles in adolescent adjustment. In another study, it was found that high school wellbeing promoted subsequent higher academic performance through better quality of interpersonal relationships (Kiuru et al., 2020). Different studies have focused on the relationship between inclusion and quality of life, stating Gaydarov (2014) in his meta-analysis, that if the effectiveness of inclusive education is to be increased, individual psychological wellbeing must also be improved. Another study show how discrimination and a lower sense of belonging increase the risk of suicidal ideas and attempts in discriminated adolescents, showing the enormous importance that inclusion and the school system can have on the wellbeing of young people, although it is not clear how these variables of discrimination, inclusion and wellbeing are related (Boyd et al., 2024). And finally, various reviews have shown the negative impact of discrimination on the quality of life in young people (Civitillo et al., 2023; Mendoza et al., 2024). However, despite the existence of the aforementioned studies, there is currently no published research that shows the effects of discrimination on the quality of life or wellbeing of Spanish adolescents and the role that inclusive education plays in this context through a model that relates these variables. Therefore, the present study contributes to the literature by examining the effects of perceived discrimination on quality of life while evaluating the mediating role of perceived school inclusion within a nationally representative Spanish sample.

Considering the aforementioned, the aim of this study is to investigate the effects of discrimination on adolescents’ quality of life, including examining the role of inclusive education in this relationship. To this end, a structural equation model is developed, which relates these variables in a sample of Spanish adolescents.

## 2 Materials and methods

### 2.1 Participants

Although the initial sample included 1,000 adolescents, a rigorous data screening process was applied to ensure data quality. First, 81 multivariate outliers were identified using Mahalanobis distance (D^2^) at the significance level *α* = 0.001 (Hair et al., 2019), with the maximum value being 23.97. Second, Guttman errors were calculated to identify atypical response patterns (e.g., straight-lining), leading to the exclusion of 8 cases. Finally, 72 participants were excluded due to incomplete responses on one or more of the key study variables (EDS, PIQ, or KINDL). This resulted in a final total of 839 participants included in the analysis. In this final sample, 50% identified as female, 49.3% as male, and 0.7% chose not to disclose their gender, with ages ranging from 12 to 16 years (Mean = 14; Standard Deviation = 1.41). The participants were selected using a method of stratified random sampling according to age, gender and national distribution (sampling error 3.1% with a confidence level of 95.5% for an infinite population and assuming maximum uncertainty). The distribution of the sample across Spain was representative, although the vast majority (92.8%) identified their ethnicity as Western European, excluding other categories such as Roma, Eastern European, or Afro-descendants. The majority of adolescents attended public schools (69.5%), with fewer attending charter schools (25.6%) and/or private schools (4.9%). Moreover, only 7.3% of the participants reported having a diagnosis of mental disorder, physical illness, or disability.

### 2.2 Procedure

An online survey was conducted using the CAWI (Computer-Assisted Web Interview) method via an access panel provided by Analysis and Investigation company. Data collection occurred during October and November 2023. Participants were required to meet specific criteria: (a) aged between 12–16 years, (b) access to the internet and a mobile device or computer. Participants were invited to participate through personalized links, targeting parents or legal guardians of children aged 12 to 16. They received information about the study and provided informed consent before participating. Subsequently, children completed the survey using multi-device technology. A cross-sectional natural group design (NGD) was employed to ensure response consistency. The questionnaire comprised two parts: the first part, directed at families, included 6 closed-ended questions to gather socio-demographic data, followed by questions for adolescents. The average completion time was 5 min for adults and 25 min for adolescents. All collected data are anonymous and have been approved by the ethics commission of the Ethics Commission of the University of Valladolid (protocol code PI23-3245NOHCUV).

### 2.3 Variables and instruments

Socio-demographic characteristics were assessed through targeted questions. Parents provided information on their child’s age, the type of educational institution they attend, and whether the child has experienced any mental disorder, physical illness, or disability. Adolescents were asked to provide their gender and ethnicity.

Discrimination was assessed using the Everyday Discrimination Scale (EDS; Williams et al., 1997) in its Spanish adaptation (Campos et al., 2016; Flores et al., 2023; Miguel-Alvaro et al., 2025). This scale consists of 9 items that explore common experiences of perceived discrimination, with statements such as “You receive less respect than others” or “People behave as if they are superior to you.” In the version administered in this study, responses were recorded on a 6-point frequency scale, with the following anchors: 1 = Almost every day; 2 = At least once a week; 3 = A few times a month; 4 = A few times a year; 5 = Less than once a year; 6 = Never. For interpretability, responses were recoded after data collection so that higher scores reflected greater perceived discrimination (i.e., Never = 0 … Almost every day = 5), and these recoded variables were used in all subsequent analyses. The EDS does not include reverse-keyed items; therefore, this procedure represents a directional recoding of the response scale, rather than item-level reverse scoring. In the present sample, the scale showed high reliability (ordinal *α* = 0.89; ωt = 0.89).

To evaluate QoL, the Questionnaire for Measuring Health-Related Quality of Life in Children and Adolescents (KINDL-R) (Bullinger et al., 2008; Ravens-Sieberer and Bullinger, 1998) was used in its Spanish version (Rajmil et al., 2004). This instrument has shown sufficient evidence of both reliability and validity in the interpretation of its scores and has been used in various studies within the Spanish context (Urzúa and Mercado, 2008; Fernández-López et al., 2004). The test is designed to be used with populations aged 8 to 16 and consists of 24 items distributed across six subscales: (1) Physical Wellbeing; (2) Emotional Wellbeing; (3) Self-esteem; (4) Family Wellbeing; (5) Friends; (6) School. KINDLR responses are collected on a five-category Likert scale ranging from “0 = never” to “4 = always.” Following the recommendation of the KINDL authors, all raw scores were transformed to a 0–100 scale to facilitate interpretation and comparability. The transformation was performed using the standard formula:


$$\text{Transformed score}=(\begin{array}{l} \left(\begin{array}{l} \mathrm{Raw}\;\text{score}\\ -\text{Minimum possible score} \end{array}\right)\\ /(\begin{array}{l} \text{Maximum possible score}\\ -\text{Minimum possible score} \end{array}) \end{array})\times 100$$

Higher scores indicate better perceived QoL. In this sample, the scale No showed high reliability (ordinal *α* = 0.90; ωt = 0.90).

Perceptions of inclusion were evaluated using the Perceptions of Inclusion Questionnaire (PIQ) (Venetz et al., 2015) in its Spanish version (PIQ-E) (Rodríguez-Medina et al., 2024). This instrument measures adolescents’ subjective perceptions across three dimensions: Emotional and Social Inclusion (EMI), Sense of Inclusion (SOI), and Academic Self-Concept (ASC). Each subscale comprises 4 items rated on a 4-point agreement scale (1 = strongly disagree; 2 = disagree; 3 = agree; 4 = strongly agree), with items 4, 8, and 12 being reverse-scored. Sample items from the EMI subscale include statements like “I enjoy attending school” or “I find school enjoyable.” From the SOI subscale: “I have many friends in my class” or “I get along well with my classmates.” And from the ASC subscale: “I perform well academically” or “I find many aspects of school challenging.” Reliability was assessed for each subscale, demonstrating adequate internal consistency: Emotional Inclusion (α = 0.89; ω_t_ = 0.90), Social Inclusion (α = 0.91; ω_t_ = 0.92), and Academic Self-Concept (α = 0.84; ω_t_ = 0.85). The overall scale also demonstrated high reliability, with ordinal alpha (α = 0.91) and omega (ω_t_ = 0.91).

### 2.4 Data analysis

In the first phase, a descriptive analysis of the entire dataset was performed. In the second stage, the measurement models for all instruments were evaluated using Confirmatory Factor Analysis (CFA) using the lavaan (Rosseel, 2014) package in R. The statistical significance and magnitude of factor loadings were assessed to confirm that items adequately reflected their corresponding latent constructs. During this phase, evidence regarding convergent and discriminant validity as well as reliability was obtained for all instruments. To accomplish this, each model was estimated based on prior literature (Bullinger et al., 2008; Ravens-Sieberer and Bullinger, 1998; Venetz et al., 2015). In the third phase, gender invariance of the structural model was examined. The progressive estimation of invariance began with the baseline model (configural invariance) and continued with metric (weak), scalar (strong), and strict invariance levels (Liu et al., 2017). Following the procedure recommended by Liu et al. (2017) for testing invariance with ordinal data, we also evaluated the practical significance of any invariance violations through sensitivity analyses.

In the fourth phase, to explore the relationship between discrimination and QoL in adolescents, a Structural Equation Model (SEM) was estimated using the lavaan package (Rosseel, 2014) in R. Model fit was evaluated using multiple indices, including the chi-square test, CFI, TLI, RMSEA, and SRMR. Following established recommendations, CFI and TLI values ≥ 0.95 were interpreted as good fit (≥ 0.90 acceptable), RMSEA values ≤ 0.06 indicated good fit (≤ 0.08 acceptable), and SRMR values ≤ 0.08 reflected adequate fit (Hu and Bentler, 1999). Standardized path coefficients were examined to determine the strength and significance of the direct and indirect effects between inclusion and QoL. Mediation analyses were conducted to investigate potential mediating and moderating variables that could influence the relationship between the two constructs.

## 3 Results

The results show discrimination scores below the mean of the questionnaire (*M* = 45.29; SD = 8.14), revealing that the sample of adolescents does not suffer discrimination frequently, with the most common forms of discrimination being feeling undervalued by others, being treated worse or being called names or insulted. On the other hand, the sample reveals medium-high levels of quality of life (*M* = 68.95; SD = 12.61), where in general perceptions of wellbeing are adequate. In relation to inclusion, the results show that it is also medium-high (*M* = 68.01; SD = 15.59), especially in the social dimension and relationships with peers. Details on the total scores as well as on the individual items of each scale can be found in the Supplementary material.

### 3.1 Measurement models

#### 3.1.1 Everyday discrimination scale

The CFA confirmed that all items in this scale fit well within a single-factor model, meaning they all contribute to measuring everyday discrimination. The model showed a good fit to the data (*χ*^2^(27) = 48.144, RMSEA = 0.031, CFI = 0.993, TLI = 0.991, and SRMR = 0.046), indicating that the scale is reliable for assessing discrimination experiences among adolescents (Table 1).

**Table 1:** Standardized factor loadings for the everyday discrimination scale.

Indicator	*B*	SE	*Z*	Beta	*R* ^2^
DISCRI1r	0.72	0.03	28.07	0.68	0.46
DISCRI2r	0.81	0.03	28.67	0.75	0.56
DISCRI3r	0.45	0.02	22.02	0.53	0.28
DISCRI4r	0.76	0.03	26.46	0.66	0.43
DISCRI5r	0.33	0.02	17.16	0.43	0.18
DISCRI6r	0.58	0.02	24.52	0.63	0.40
DISCRI7r	0.86	0.03	28.04	0.63	0.40
DISCRI8r	0.60	0.03	23.84	0.58	0.34
DISCRI9r	0.44	0.02	23.55	0.59	0.35

All *p*-values were statistically significant at *p* < 0.001.

#### 3.1.2 PIQ questionnaire

The confirmatory factor analysis (CFA) confirmed that the PIQ questionnaire follows a three-factor structure, representing Emotional Inclusion, Social Inclusion, and Academic Self-Concept. The model demonstrated a good fit to the data (*χ*^2^(51) = 139.609, RMSEA = 0.042, CFI = 0.988, TLI = 0.984), confirming its validity (Table 2).

**Table 2:** Standardized factor loadings for the PIQ questionnaire.

Latent factor	Indicator	*B*	SE	*Z*	Beta	*R* ^2^
Emotional	PIC1	0.666	0.022	30.764	0.842	0.709
	PIC4R	0.582	0.033	17.862	0.650	0.423
	PIC7	0.639	0.022	28.410	0.818	0.669
	PIC10	0.526	0.024	22.357	0.781	0.611
Social	PIC2	0.576	0.023	24.578	0.777	0.603
	PIC5	0.484	0.019	26.021	0.806	0.650
	PIC8R	0.491	0.024	20.463	0.702	0.493
	PIC11	0.486	0.019	25.918	0.817	0.668
Academic	PIC3	0.493	0.021	23.007	0.747	0.558
	PIC6	0.498	0.024	21.064	0.694	0.481
	PIC9	0.454	0.022	20.665	0.734	0.538
	PIC12R	0.455	0.031	14.567	0.583	0.340

All p-values were statistically significant at *p* < 0.001.

#### 3.1.3 Questionnaire for measuring health-related quality of life in children and adolescents (KINDL-R)

The CFA confirmed that the six-factor structure provided an acceptable fit to the data (*χ*^2^(237) = 1268.128; RMSEA = 0.063; CFI = 0.961; TLI = 0.954), supporting its validity. Results can be seen in Table 3.

**Table 3:** Standardized factor loadings for the KINDL-R questionnaire.

Latent factor	Indicator	*B*	SE	*Z*	Beta	*R* ^2^
FI	KINDL1r	0.45	0.03	16.13	0.49	0.24
	KINDL2r	0.42	0.03	13.50	0.48	0.23
	KINDL3r	0.49	0.03	15.89	0.55	0.31
	KINDL4	0.61	0.03	22.43	0.74	0.55
EM	KINDL5	0.47	0.02	19.76	0.61	0.38
	KINDL6r	0.46	0.03	15.79	0.55	0.30
	KINDL7r	0.64	0.03	22.71	0.67	0.45
	KINDL8r	0.56	0.03	19.44	0.58	0.34
AU	KINDL9	0.73	0.03	29.12	0.81	0.66
	KINDL10	0.76	0.03	29.48	0.82	0.68
	KINDL11	0.72	0.02	30.23	0.90	0.81
	KINDL12	0.46	0.03	16.25	0.59	0.34
FA	KINDL13	0.57	0.02	26.24	0.77	0.59
	KINDL14	0.63	0.02	25.71	0.82	0.68
	KINDL15r	0.59	0.03	21.15	0.66	0.44
	KINDL16r	0.71	0.03	24.39	0.72	0.52
AM	KINDL17	0.52	0.03	18.73	0.58	0.34
	KINDL18	0.52	0.02	22.68	0.74	0.54
	KINDL19	0.51	0.02	22.99	0.74	0.55
	KINDL20r	0.69	0.03	22.23	0.66	0.43
ES	KINDL21	0.55	0.03	20.48	0.69	0.47
	KINDL22	0.53	0.03	17.33	0.55	0.30
	KINDL23r	0.14	0.04	3.33	0.13	0.02
	KINDL24r	0.45	0.04	11.75	0.41	0.17

All *p*-values were statistically significant at *p* < 0.001.

#### 3.1.4 Inclusion as mediator between discrimination and quality of life

**Figure 1:**
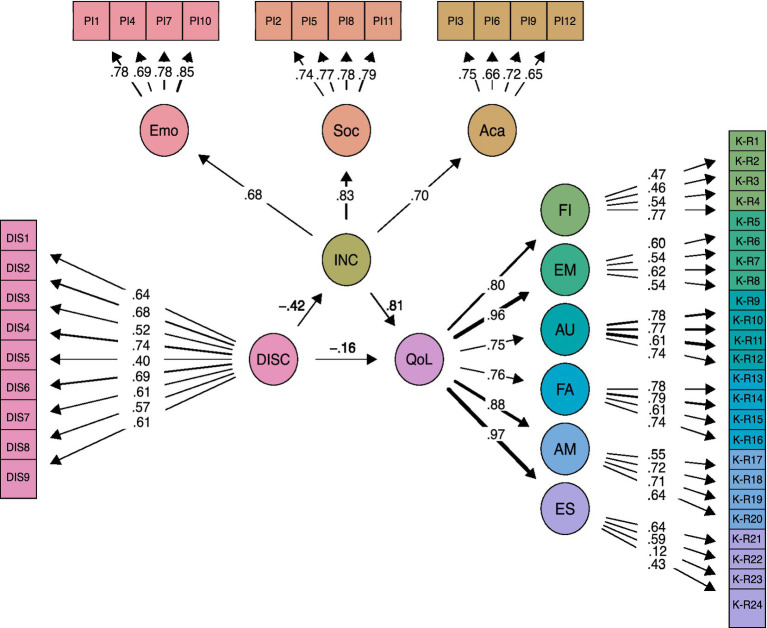
Hypothesized structural model. Structural equation model testing the mediating role of perceived school inclusion (INC) in the relationship between perceived discrimination (DISC) and quality of life (QoL). Inclusion is composed of emotional (Emo), social (Soc), and academic (Aca) inclusion. Quality of life is measured across six dimensions: physical wellbeing (FI), emotional wellbeing (EM), autonomy (AU), family relationships (FA), friendships (AM), and school environment (ES). Observed variables and standardized factor loadings are shown. All coefficients are standardized and statistically significant (*p* < 0.001).

The relationship between Inclusion, Discrimination and Quality of Life was studied using SEM. Figure 1 presents the final proposed model, in which discrimination negatively affects both inclusion and QoL. Discrimination directly and negatively affects quality of life, while it is also affected indirectly through inclusion, which acts as a mediator between the two variables. More discrimination is related to lower quality of life, while more discrimination implies less inclusion, and less inclusion is related to lower quality of life. In addition, inclusion shows a strong positive association with quality of life, indicating that a greater sense of belonging contributes to improved wellbeing.

Standardized path coefficients further confirmed that Discrimination significantly reduces both Inclusion (*β* = −0.457, *p* < 0.001) and QoL (*β* = −0.436, *p* < 0.001), whereas Inclusion has a strong positive impact on QoL (*β* = 1.575, *p* < 0.001). The indirect effect of Discrimination on QoL through Inclusion was significant (*β* = −0.719, *p* < 0.001), confirming that Inclusion partially mediates the negative impact of Discrimination on adolescent wellbeing. Results in detail can be seen in Table 4.

**Table 4:** Standardized path coefficients of the mediation model.

Path	Estimate	S.E.	ci. lower	ci. upper	Std	*z*	*p*
QoL ~ DISCRI	−0.436	0.076	−0.500	−0.201	−0.163	−4.592	<0.001
INC ~ DISCRI	−0.457	0.048	−0.552	−0.362	−0.415	−9.445	<0.001
QoL ~ INC	1.575	0.174	1.234	1.915	0.805	9.062	<0.001
Indirect effects	−0.719	0.110	−0.935	−0.503	−0.334	−6.539	<0.001

This final mediated model was compared to another model where the relationship of Discrimination and QoL was not mediated by Inclusion, this model was called ‘Alternative Model’. In this model, it is shown that although inclusion partially buffers the negative impact of discrimination, discrimination exerts a direct effect on quality of life resulting in a model that is sufficient in terms of its parameter outcomes. But the results show a worse fit of the alternative model compared with the final one, showing how introducing the mediation of Inclusion improves the statistics, thus underlining the importance of Inclusion. The final mediated model highlights the crucial role of school belonging in mitigating the effects of Discrimination and promoting QoL. Table 5 displays the fit indices of both models.

**Table 5:** Fit indices of the proposed model.

Model	*χ*^2^ (df)	*p(χ^2^)*	RMSEA	SRMR	CFI	TLI
Mediated model	2782.79 (933)	< 0.001	0.049	0.063	0.965	0.963
Alternative model	2872.73 (934)	< 0.001	0.050	0.076	0.964	0.961

## 4 Discussion

The results of this study highlight the significant relationship between perceptions of inclusion and QoL in adolescents, reinforcing previous findings on the impact of inclusive education (Gaydarov, 2014). Our data show that a greater perception of inclusion correlates with a higher quality of life, supporting the idea that a sense of belonging and acceptance within the school environment is essential for adolescents’ overall wellbeing. These findings are consistent with those of Arslan (2021), who observed that school belonging is positively associated with adolescents’ subjective wellbeing and fewer mental health issues.

A key aspect of this study concerns the role of perceived inclusion as a mediating factor between discrimination and quality of life. Our findings indicate that although discrimination has a direct negative impact on quality of life, as shown in other studies (Gaydarov, 2014), this effect is partially mediated by perceived inclusion, which mitigates some of its negative consequences. This suggests that the benefits of inclusion are partly explained by the negative impact of discrimination, as also indicated by Coley et al. (2017), who found that perceived discrimination was associated with lower quality of life in older African American women. Similarly, the study by Sevillano et al. (2014) in an immigrant population in Spain reinforces the view that perceived discrimination is a key predictor of physical and mental health.

Our model presents a comprehensive view of the interconnection between Discrimination, Inclusion, and QoL, a pattern supported in previous research, such as the study by Kakemam et al. (2024). This latter work examines how social factors, such as social support, governance, and perceived discrimination, affect mental health in Iranian adults, identifying the mediating role of quality of life in this relationship. Although Kakemam et al. (2024) emphasize the importance of quality of life as a mediator in the connection between social factors and mental health disorders, their focus is directed toward an adult population and centers primarily on the effects of social determinants on mental health disorders.

The findings of our study align with broader theoretical frameworks on inclusion and mental health (Gómez et al., 2021; Schalock et al., 2016; Verdugo et al., 2021), including the Quality of Life and Supports Model (MOCA) by Verdugo et al. (2021), which provides an operational integration of the quality of life and support paradigms for people with intellectual disabilities. This model emphasizes an inclusive environment as a means to improve wellbeing. Our study highlights the importance of fostering inclusive practices not only to improve direct outcomes such as academic performance (Kart and Kart, 2021) but also to address indirect factors that can impact adolescents’ development. For instance, Montoro et al. (2021) found that school belonging, a key indicator of academic wellbeing, is negatively affected by discrimination from both adults and peers, impacting students’ sense of belonging.

However, our results also reveal that inclusion, while beneficial, may not fully counteract the negative effects of discrimination on quality of life among diverse adolescent populations. Our findings show that while inclusion directly enhances quality of life, adolescents who experience discrimination—particularly those from minority backgrounds or those facing physical or mental challenges—report lower quality of life. These findings are similar to the research by Sellers et al. (2013) and Nascimento et al. (2020), who highlighted the compounded effects of discrimination on the wellbeing of marginalized youth. The broader social context also plays a significant role. Thomas (2013) suggests that to maximize the positive impact of inclusive education, it is crucial to rethink traditional approaches to students’ difficulties in school and to recognize how factors such as inequality and social judgment contribute to learning barriers.

Some limitations of this research refer to the characteristics of the sample, which, although it has ensured a balanced sample in terms of gender, age and territorial distribution in Spain, there is a lack of access to specific populations, such as adolescents of very low and high socio-economic status, or truly rural populations, probably due to the online methodology employed. In addition, the ethnic composition of the sample was highly homogeneous, with 92.8% of participants identifying as Western European. This limited diversity may constrain the generalizability of the findings, particularly regarding the experiences of adolescents from minoritized ethnic backgrounds. Future research should include more ethnically diverse samples to enhance external validity. On the other hand, it is necessary to take into account the limitations of the cross-sectional methodology and structural equations. Although they are advanced and complete analyses, they still provide a static picture of reality, making it difficult to make absolute statements when formulating explanatory theories of phenomena as complex as the relationships between the variables presented in this study. Complementing this model in the future with longitudinal and qualitative studies will undoubtedly enrich knowledge in the area. Along the same lines, the replicability of this model in other samples and cultures would ensure the generalisability of the results obtained, which until then should be interpreted with caution.

The conclusions of this study underscore the significant relationship between inclusion and quality of life (QoL) in adolescents, highlighting that the perception of inclusion is not only directly associated with improved quality of life but also appears to mediate experiences of discrimination, which negatively impact adolescent wellbeing. These results are consistent with previous research suggesting that a sense of belonging and acceptance within the educational environment is essential for positive development during adolescence.

Our findings confirm that discrimination has a persistent negative impact on adolescent wellbeing. While inclusion mitigates some of its effects, targeted interventions against discrimination remain essential to improve quality of life. This finding suggests that, to maximize the benefits of inclusive practices, effective policies should be in place to address and mitigate discrimination. In this regard, the findings support the need to promote an educational environment that is not only inclusive but also actively committed to preventing and reducing discrimination against adolescents from diverse backgrounds and characteristics. These findings reinforce the importance of fostering inclusive school environments not only as a means of improving adolescent wellbeing, but also as a lever for driving meaningful educational change toward greater equity and social justice.

## Data Availability

The raw data supporting the conclusions of this article will be made available by the authors, without undue reservation.
