# Supplementary material for: Discrimination, school inclusion, and quality of life in adolescence: a mediation analysis
Source: Front Psychol. 2026 Jan 12;16:1722505. doi: 10.3389/fpsyg.2025.1722505 (PMC12833353; doi:10.3389/fpsyg.2025.1722505)
Supplement: Supplementary file 1 [file Table_1.docx]

Supplementary Material

1. **Table s1**

*Everyday Discrimination Scale Descriptives*

| **Item** | **n** | **mean** | **sd** | **median** | **skew** | **kurtosis** | **se** |
| --- | --- | --- | --- | --- | --- | --- | --- |
| You are treated with less courtesy or politeness than other people. | 839 | 0.87 | 1.06 | 1 | 1.17 | 0.93 | 0.04 |
| You are treated with less respect than other people. | 839 | 0.88 | 1.08 | 0 | 1.04 | 0.33 | 0.04 |
| You receive worse service than others in a restaurant or a store. | 839 | 0.47 | 0.85 | 0 | 1.67 | 1.66 | 0.03 |
| People act as if they think you are not intelligent. | 839 | 0.91 | 1.16 | 0 | 1.17 | 0.61 | 0.04 |
| People act as if they are afraid of you. | 839 | 0.35 | 0.78 | 0 | 2.28 | 4.38 | 0.03 |
| People act as if they think you are dishonest or untrustworthy. | 839 | 0.59 | 0.92 | 0 | 1.59 | 2.13 | 0.03 |
| People act as if they are superior to you. | 839 | 1.6 | 1.36 | 2 | 0.47 | -0.47 | 0.05 |
| You are called names or insulted. | 839 | 0.89 | 1.03 | 1 | 1 | 0.47 | 0.04 |
| You are threatened or assaulted. | 839 | 0.44 | 0.74 | 0 | 1.61 | 1.88 | 0.03 |
| TOTAL | 839 | 45.29 | 8.14 | 47 | -1.27 | 1.59 | 2.25 |

Descriptive statistics for the Everyday Discrimination Scale, which assesses adolescents' perceptions of discriminatory experiences in their daily lives can be seen in Table 1. The highest mean score corresponds to the item "People act as if they are better than you" (M = 1.6, SD = 1.36), indicating that this form of discrimination is perceived as more frequent among participants. Conversely, the item "People act as if they are afraid of you" has the lowest mean (M = 0.35, SD = 0.78), suggesting that this type of discrimination is less commonly reported. Skewness and kurtosis values reveal varying distributions, with items such as "You are treated with less courtesy" and "You receive worse service in restaurants or stores" showing right-skewed distributions, reflecting that most participants reported these experiences infrequently.

1. **Table s2**

*KINDL-R Descriptives*

| **vars** | **n** | **mean** | **sd** | **median** | **skew** | **kurtosis** | **se** |
| --- | --- | --- | --- | --- | --- | --- | --- |
| KINDL1r | 839 | 4.11 | 0.88 | 4 | -0.6 | -0.35 | 0.03 |
| KINDL2r | 839 | 3.59 | 0.83 | 3 | 0.23 | -0.26 | 0.03 |
| KINDL3r | 839 | 3.38 | 0.83 | 3 | 0.01 | 0.26 | 0.03 |
| KINDL4 | 839 | 3.72 | 0.76 | 4 | -0.18 | -0.31 | 0.03 |
| KINDL5 | 839 | 3.92 | 0.72 | 4 | -0.06 | -0.65 | 0.02 |
| KINDL6r | 839 | 3.49 | 0.76 | 3 | 0.29 | -0.02 | 0.03 |
| KINDL7r | 839 | 4.04 | 0.85 | 4 | -0.41 | -0.55 | 0.03 |
| KINDL8r | 839 | 3.87 | 0.89 | 4 | -0.27 | -0.51 | 0.03 |
| KINDL9 | 839 | 3.75 | 0.84 | 4 | -0.23 | -0.12 | 0.03 |
| KINDL10 | 839 | 3.81 | 0.85 | 4 | -0.4 | 0.18 | 0.03 |
| KINDL11 | 839 | 3.96 | 0.74 | 4 | -0.39 | 0.26 | 0.03 |
| KINDL12 | 839 | 3.51 | 0.76 | 3 | 0.34 | -0.21 | 0.03 |
| KINDL13 | 839 | 4.21 | 0.67 | 4 | -0.4 | -0.35 | 0.02 |
| KINDL14 | 839 | 4.43 | 0.69 | 5 | -0.91 | 0.04 | 0.02 |
| KINDL15r | 839 | 4.19 | 0.84 | 4 | -0.68 | -0.36 | 0.03 |
| KINDL16r | 839 | 3.67 | 0.92 | 4 | -0.05 | -0.6 | 0.03 |
| KINDL17 | 839 | 3.96 | 0.83 | 4 | -0.49 | 0.12 | 0.03 |
| KINDL18 | 839 | 3.99 | 0.65 | 4 | -0.12 | -0.25 | 0.02 |
| KINDL19 | 839 | 4.38 | 0.61 | 4 | -0.49 | -0.3 | 0.02 |
| KINDL20r | 839 | 3.91 | 0.96 | 4 | -0.48 | -0.42 | 0.03 |
| KINDL21 | 839 | 4.22 | 0.72 | 4 | -0.61 | 0.22 | 0.02 |
| KINDL22 | 839 | 3.64 | 0.92 | 4 | -0.29 | -0.35 | 0.03 |
| KINDL23r | 839 | 3.16 | 1.1 | 3 | -0.12 | -0.49 | 0.04 |
| KINDL24r | 839 | 3.29 | 1.07 | 3 | -0.11 | -0.38 | 0.04 |
| TOTAL | 8393 | 68.95 | 12.61 | 69.79 | -.15 | -.24 | .39 |

Table 2 provides descriptive statistics for the KINDL-R, a measure of health-related quality of life in adolescents. The highest mean scores are observed for "KINDL14" (M = 4.43, SD = 0.69) and "KINDL19" (M = 4.38, SD = 0.61), indicating a generally positive perception of well-being in these dimensions. In contrast, "KINDL23r" (M = 3.16, SD = 1.1) and "KINDL24r" (M = 3.29, SD = 1.07) report the lowest means, suggesting areas where adolescents might experience lower quality of life. Most variables exhibit a normal distribution, though "KINDL14" shows a notable negative skew (-0.91), indicating that many participants reported higher well-being levels in this domain.

1. **Table s3**

*PIQ Questionnaire Descriptives*

| **Item** | **n** | **mean** | **sd** | **median** | **skew** | **kurtosis** | **se** |
| --- | --- | --- | --- | --- | --- | --- | --- |
| I like going to school. | 839 | 2.94 | 0.74 | 3 | -0.45 | 0.12 | 0.03 |
| I have many friends in my class. | 839 | 3.14 | 0.69 | 3 | -0.5 | 0.19 | 0.02 |
| I learn quickly. | 839 | 3.12 | 0.62 | 3 | -0.14 | -0.25 | 0.02 |
| I don’t feel like going to school. | 839 | 2.92 | 0.85 | 3 | -0.36 | -0.58 | 0.03 |
| I get along very well with my classmates. | 839 | 3.32 | 0.55 | 3 | -0.13 | -0.17 | 0.02 |
| I am capable of solving very difficult exercises. | 839 | 2.92 | 0.68 | 3 | -0.12 | -0.31 | 0.02 |
| I like school. | 839 | 2.95 | 0.74 | 3 | -0.43 | 0.1 | 0.03 |
| I feel lonely in my class. | 839 | 3.39 | 0.63 | 3 | -0.57 | -0.48 | 0.02 |
| I am a good student. | 839 | 3.3 | 0.58 | 3 | -0.28 | 0.23 | 0.02 |
| I have fun at school. | 839 | 3.08 | 0.63 | 3 | -0.38 | 0.71 | 0.02 |
| I have a good relationship with my classmates. | 839 | 3.35 | 0.54 | 3 | -0.03 | -0.84 | 0.02 |
| For me, many things are very difficult at school. | 839 | 2.77 | 0.75 | 3 | -0.18 | -0.29 | 0.03 |
| TOTAL | 839 | 68.01 | 15.59 | 66.66 | -.026 | -.14 | 0.49 |

Table 3 summarizes the descriptive statistics for the Perceptions of Inclusion Questionnaire (PIQ), which evaluates students' sense of belonging in the school environment. The highest mean scores appear in items related to peer relationships, such as "I get along well with my classmates" (M = 3.35, SD = 0.54) and "I have many friends in my class" (M = 3.14, SD = 0.69), suggesting a generally positive perception of social inclusion. On the other hand, items reflecting negative school experiences, such as "Many things are very difficult for me at school" (M = 2.77, SD = 0.75), indicate that some students perceive academic challenges. The distribution of responses suggests a relatively balanced perception of inclusion, with minor variations across items.
